# Patient Body Weight-Tailored Contrast Medium Injection Protocol for the Craniocervical Vessels: A Prospective Computed Tomography Study

**DOI:** 10.1371/journal.pone.0088867

**Published:** 2014-02-18

**Authors:** Rebecca Kessler, Katrin Hegenscheid, Steffen Fleck, Alexander Khaw, Michael Kirsch, Norbert Hosten, Sönke Langner

**Affiliations:** 1 Institute of Diagnostic Radiology and Neuroradiology, University Medicine Greifswald, Greifswald, Germany; 2 Department of Neurosurgery, University Medicine Greifswald, Greifswald, Germany; 3 Department of Neurology, University Medicine Greifswald, Greifswald, Germany; Cuban Neuroscience Center, Cuba

## Abstract

**Objectives:**

To evaluate body weight-tailored contrast medium (CM) administration for computed tomography angiography (CTA) of the craniocervical vessels.

**Methods:**

Institutional review board approval was obtained, and all patients gave written informed consent. Sixty patients were consecutively assigned to one of three dose groups (20 patients per group) with CM doses of Visipaque 270® (iodixanol 270 mg/ml) tailored to body weight at doses of 1.5, 1.0, or 0.5 ml/kg. Region-of-interest (ROI) analysis of maximum enhancement (ME) was conducted, and signal-to-noise-ratios (SNR) and contrast-to-noise-ratios (CNR) were calculated. Retrospective comparison was performed with three matched control groups examined with a standard CM dose (80 ml of Visipaque 270®). Image quality was rated by two neuroradiologists blinded to the CM dose used. Interrater reliability was calculated using kappa statistics.

**Results:**

Body weight/BMI and ME were inversely correlated in the three control groups receiving the standard dose (r = −0.544/−0.597/−0.542/r = −0.358/r = −0.424/r = −0.280). Compared to standard dose, 1.5 ml/kg produced higher ME, SNR, and CNR in the anterior circulation (p≤0.038), 1.0 ml/kg had higher ME in cervical and medium-sized cerebral arteries (p≤0.034), and 0.5 ml/kg had lower ME, SNR and CNR for medium-sized cerebral arteries (p≤0.049). ME, SNR, and CNR were the same for 1.5 ml/kg and 1.0 ml/kg (p≥0.24), and both had higher values compared to 0.5 ml/kg (p≤0.043/p≤0.028). In patients with BMI>25, 1.5 ml/kg and 1.0 ml/kg produced higher ME than standard dose (p<0.001/p = 0.008), but ME in patients with BMI>25 did not differ between group 1 and group 2 (p = 0.673). In patients with BMI≤25, 1.5 ml/kg and 1.0 ml/kg produced ME comparable to standard dose (p = 0.132/p = 0.403). Regardless of patient weight, 0.5 ml/kg yielded lower ME than standard dose (p = 0.019/0.002).

**Conclusions:**

Craniocervical CTA with a body weight-tailored CM dose of 1.0 ml/kg (270 mg iodine/ml) reduces iodine load in patients weighing <80 kg while producing ME similar to standard dose and improves ME in patients with BMI>25.

## Introduction

Since the implementation of helical and multidetector computed tomography (MDCT), computed tomography angiography (CTA) has become a widely accepted technique for the assessment of the craniocervical vessels. It is often used for initial neurovascular imaging because it allows rapid evaluation of a wide range of craniocervical vascular conditions including stroke, aneurysms, traumatic vessel lesions, and vascular malformations [1]–[3].

CTA requires the administration of iodinated contrast medium (CM). Disadvantageously, iodinated CM can cause contrast-induced nephropathy (CIN), especially in patients with pre-existing renal impairment [4]. Concomitant risk factors are diabetic nephropathy, dehydration, congestive heart failure, concurrent administration of nephrotoxic drugs, and the dose and type of CM [1]. CIN ranks third among the causes of hospital-acquired acute renal failure in the US [2].

The policy of many radiology departments is to administer a uniform dose of CM to all patients undergoing CTA of the craniocervical vessels. However, the degree of contrast enhancement achieved is strongly dependent on the amount of CM injected in relation to the patient's body weight. Several studies have investigated this relationship, indicating that, for protocols with a fixed contrast dose, there is an inverse correlation between body weight and vascular or parenchymal contrast enhancement in abdominal enhanced computed tomography (CT) [3] and pulmonary CTA [4]–[6].

Hence, some authors have suggested contrast injection protocols with iodine doses tailored to patient body weight for abdominal enhanced CT [7]–[10], pulmonary CTA [11], and coronary CTA [12]–[15]. Awai et al. [16] report having achieved almost constant aortic enhancement irrespective of body weight when using a protocol with the dose tailored to patient weight and a fixed injection duration.

These findings are good reason to evaluate the effect of body weight-tailored CM dosage for CTA of the craniocervical vessels. Unlike for imaging of the abdomen, pulmonary vessels and coronary arteries, data on weight-tailored CTA of the craniocervical vessels are sparse.

Thus, the purpose of our study was to prospectively evaluate whether tailoring CM dose to patient body weight is also beneficial for craniocervical CTA with regard to quantitative vascular enhancement and subjective image quality and should be favored over administration of a uniform CM dose.

## Materials and Methods

### Patient Population and Ethics Statement

The study was approved by the ethics committee at the medical faculty of the University of Greifswald (registration number BB 65/09). Written informed consent was obtained from all patients. Between July 2009 and March 2010, 60 patients were enrolled in this prospective study. The patients were assigned consecutively to one of three protocols (20 patients per protocol). In all protocols the intravenous CM dose of Visipaque 270® (iodixanol 270 mg/ml, GE Healthcare Buchler, Braunschweig, Germany) was tailored to patient body weight using 1.5 ml/kg, 1.0 ml/kg and 0.5 ml/kg in groups 1, 2 and 3, corresponding to 405 mg, 270 mg and 135 mg iodine/kg body weight, respectively. Neurological or neurosurgical adult patients (≥18 years) with suspected or known cerebrovascular disease were included. Exclusion criteria were renal failure defined as glomerular filtration rate below 60 ml/min/1.73 m^2^, manifest hyperthyroidism, previous history of contrast medium intolerance, and pregnancy or lactation. We also excluded patients treated in the intensive care unit and patients with clinically apparent cardiac failure. For retrospective comparison with the standard protocol using a fixed dose of 80 ml of Visipaque 270®, three groups of patients, who had been examined between January 2008 and June 2009, were formed, referred to as controls 1, 2 and 3, respectively. To reduce demographic bias, the control patients were also selected from the neurological and neurosurgical patient population and each of the control patients was age- and sex-matched to one of the consecutive study patients. For further analysis, groups and controls were subdivided according to body mass index (BMI). A BMI of 25 was set as threshold for overweight referring to the BMI classification of the World Health Organization.

### CT Protocol

All CT studies were performed on a helical 16-slice MDCT scanner (Somatom Sensation 16, SIEMENS Medical Solutions, Erlangen, Germany). CTA was performed using semiautomatic bolus tracking in the common carotid artery at the level of the C5 vertebra with a threshold of 200 Hounsfield units (HU). The CM was administered using a power injector (MedRad Medical Systems, Volbach, Germany) with a flow rate of 4 ml/s, followed by a saline flush of 40 ml injected at the same rate. The CTA scan range covered the volume from the C7 vertebra to the vertex. Scan parameters were 16×0.75 mm collimation, pitch of 1.25, 1 mm reconstructed slice thickness, 120 mA tube current, and 100 kV tube voltage. For further analysis, thick slab maximum intensity projections (MIP) in orbitomental and coronal planes were reconstructed with 20 mm slice thickness.

### Image Analysis

Region-of-interest (ROI) analysis was used for quantitative image evaluation at a clinical workstation (Agfa IMPAX ES 5.2, Agfa HealthCare, Mortsel, Belgium). A radiologist with 3 years of experience in CTA of the craniocervical vessels manually placed a circular or elliptic ROI on transverse source images in the following predefined regions: common carotid arteries (CCA), 2 cm proximal to the bifurcation; external carotid arteries (ECA), 1 cm distal to the bifurcation; extracranial internal carotid arteries (ICAex), 2 cm below the skull base; intracranial internal carotid arteries (ICAin), immediately proximal to the internal carotid bifurcation; A1 segment of anterior cerebral arteries (ACA); anterior communicating artery (AcomA); M1 segment of middle cerebral arteries (MCA); posterior communicating arteries (PcomA); P2 segment of posterior cerebral arteries (PCA); superior cerebellar arteries (SCA); anterior inferior cerebellar arteries (AICA); basilar artery (BA), immediately distal to the confluence of the vertebral arteries; posterior inferior cerebellar arteries (PICA); and vertebral arteries (VA), 2 cm below the skull base. Wherever possible, ROIs were placed on both sides. The size of the ROI was adjusted to the vessel diameter. To reduce bias, two independent measurements were performed at each measurement site. The average of the two measurements was defined to be the maximum enhancement (ME). Congenital variations in the anatomy of the circle of Willis such as aplasia or hypoplasia of arteries or arterial segments were recorded. In case of aplastic arteries these anatomical target locations were excluded from evalutation. In case of hypoplastic arteries these were skipped if the vessel diameter was too small to allow ROI placement. Craniocervical arterial stenosis was graded according to NASCET criteria [17]. If the stenosis involved the site of measurement in a vessel, the size of the ROI was reduced to exclude the atherosclerotic plaque if the degree of stenosis was <70%. If the stenosis was >70% or in case of occlusion, the vessel was excluded from evaluation. Measurements in vessel territories distal to a stenosis or occlusion were conducted.

ROIs for measuring attenuation (HU) of background and brain parenchyma in the centrum semiovale on the right side enclosed a constant area of 2 cm^2^ in all patients. These ROIs served to calculate signal-to-noise and contrast-to-noise ratios (SNR and CNR) using the following equations:
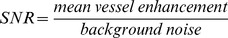


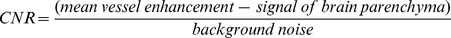



Background noise was defined as standard deviation of the measured HU of the background.

### Visual Analysis

Two board-certified neuroradiologists from our institution with more than 10 years of experience in CTA of the craniocervical vessels (S.L., 11 years; M.K., 12 years) assessed overall image quality of all 60 CTA datasets obtained with one of the three body weight-tailored protocols and of the 60 CTA datasets acquired with the standard protocol. Assessment was carried out on the MIP reconstructions. Both readers were blinded to the contrast injection protocol used. Visual image quality was rated using a 5-point visual analog scale with the following scores: 0 =  unacceptable (contrast enhancement insufficient to make a radiologic diagnosis); 1 =  poor (contrast enhancement just about acceptable to make a radiologic diagnosis); 2 =  fair (contrast enhancement sufficient for radiologic diagnosis, but image quality unsatisfactory); 3 =  good (contrast enhancement adequate and image quality satisfactory); 4 =  optimal (excellent contrast enhancement and image quality).

### Statistical Analysis

Results for ME, SNR, and CNR were expressed as medians (25th percentile–75th percentile). Multiple comparisons of ME, SNR, and CNR of the different protocol groups were performed by using the Kruskal-Wallis test. If the overall differences were statistically significant, post-hoc analysis was carried out by means of the Mann-Whitney U-test. Interim analysis by Bonferroni-Holm adjustment was conducted to counteract the problem of multiple comparisons. Normal weight (BMI≤25) and overweight (BMI>25) subgroup analysis for ME of the MCA for groups and controls was performed by using the two-tailed Student t-test. Spearman's rank correlation coefficient (r) was used to investigate the relationships between patient body weight as well as BMI and ME in the MCA in each group. The visual scores of the different protocol groups were also compared by Kruskal-Wallis test. If there was a statistically significant difference among all groups, pairwise comparisons were performed using the Mann-Whitney U-test. Interrater reliability with regard to visual analysis was assessed using kappa statistics [18]. Analyses were performed using SPSS (version 20.0; SPSS Chicago, Illinois, USA). A p-value of p<0.05 was considered statistically significant.

## Results

### Patient Population

Demographic data of the patient population and data on administered CM volumes and doses are provided in Table 1. The results for high-grade arterial stenosis and vascular occlusion are presented in Table 2. Data on congenital variations in the circle of Willis are summarized in Table 3.

**Table 1:** Patient demographics in the three study groups and in the three control groups.

	*Group 1 (1.5 ml/kg)*	*Group 2 (1.0 ml/kg)*	*Group 3 (0.5 ml/kg)*	*Control 1*	*Control 2*	*Control 3*	*p-value*
*Sex (m/f)*	9/11	12/8	9/11	9/11	12/8	9/11	.794
*Age (years)*	62 (57–69)	73 (59–78)	69 (55–76)	62 (57–69)	73 (59–78)	69 (55–76)	.379
*BW (kg)*	78 (67–88)	78 (70–88)	76 (66–85)	70 (66–84)	79 (69–94)	75 (66–85)	.789
*Height (m)*	1.70 (1.64–1.76)	1.69 (1.62–1.75)	1.66 (1.59–1.70)	1.64 (1.58–1.72)	1.70 (1.65–1.75)	1.66 (1.60–1.76)	.314
*BMI (kg/m^2^)*	26.3 (24.6–29.2)	26.1 (23.7–32.2)	28.0 (26.3–30.1)	28.0 (24.4–31.1)	27.4 (24.4–31.1)	26 (24.3–29.0)	.749
*CMV (ml)*	117 (101–137)	78 (70–88)	39 (33–43)	80	80	80	
*TID (mg)*	31 455 (27 135–36 855)	21 060 (18 900–23 625)	10 395 (8 978–11 610)	21 600	21 600	21 600	
*RID (mg/kg BW)*	405	270	135	305 (255–322)	274 (230–316)	288 (256–329)	

Data on age, BW, height, BMI, CMV, TID, and RID are presented as medians (25th percentile–75th percentile); BW – body weight; BMI – body mass index; CMV – contrast medium volume; TID – total iodine dose; RID – relative iodine dose. Overall p-value was calculated by Kruskal-Wallis test.

**Table 2:** Vascular occlusions and high-grade vascular stenoses (≥70% NASCET).

	CCA		ICA ex		ECA		ICA in		MCA		ACA		PCA		VA		BA
	L	R	L	R	L	R	L	R	L	R	L	R	L	R	L	R	
Group 1	1	1	1	3	1	1	1	3	0	1	0	0	0	0	1	1	0
Group 2	0	0	1	0	0	0	1	0	0	0	0	0	1	0	0	0	0
Group 3	0	0	0	1	0	0	0	0	0	0	0	0	0	0	0	1	0
Control 1	0	0	1	1	0	0	1	2	0	0	0	0	0	0	0	0	1
Control 2	0	0	2	0	0	0	1	0	0	0	0	0	1	0	0	0	1
Control 3	0	0	2	0	0	0	1	0	1	0	0	0	0	0	0	0	1

Data are given as total numbers of patients per group with vessel occlusion or high-grade vessel stenosis. A high-grade stenosis was defined as ≥70% luminal narrowing according to the NASCET classification. Data are subdivided by side of location of stenosis or occlusion. L – left side, R – right side.

**Table 3:** Congenital arterial variants.

	*A1-segment*		*AComA*	*PComA*		*SCA*		*AICA*		*PICA*		*VA*	
	*L*	*R*		*L*	*R*	*L*	*R*	*L*	*R*	*L*	*R*	*L*	*R*
*Group 1*	1	2	0	7	5	0	0	4	3	0	0	1	0
*Group 2*	0	1	0	5	7	0	0	2	3	2	1	0	0
*Group 3*	2	0	4	7	9	1	1	8	9	3	4	0	0
*Control 1*	0	2	6	10	6	0	0	6	8	2	3	0	1
*Control 2*	1	2	5	9	4	0	0	9	9	5	6	0	0
*Control 3*	0	0	2	8	7	0	0	5	2	2	1	0	0

Data are given as total number of patients per group with arterial aplasia or marked hypoplasia. Data are subdivided by side of location of aplasia/hypoplasia. L – left side, R – right side.

### Quantitative Assessment

Tables 4, 5 and 6 present the results of quantitative analysis for ME, SNR, and CNR by craniocervical vascular territory and contrast protocol group including p-values for multiple and paired comparisons. The multiple comparison test revealed statistically significant differences between the three body weight-tailored groups for ME, SNR, and CNR in all vascular territories (Tables 4, 5 6). In the control population, there were no statistically significant differences between the groups (Table 4).

**Table 4:** Maximum enhancement (ME) in HU of the craniocervical vessels in body weight-tailored groups and controls.

				*Multiple comparison (p-value)*				*Multiple comparison (p-value)*	*Paired comparison (p-value)*					
*Vascular territory*	*Group 1 (1.5 ml/kg)*	*Group 2 (1.0 ml/kg)*	*Group 3 (0.5 ml/kg)*	*Groups*	*Control 1*	*Control 2*	*Control 3*	*Controls*	*Group 1/Group 2*	*Group 1/Group 3*	*Group 2/Group 3*	*Group1/Control 1*	*Group 2/Control 2*	*Group 3/Control 3*
*CCA*	371 (340–473)	435 (353–464)	354 (259–385)	.001**	345 (284–452)	363 (312–434)	376 (321–440)	.550	.783	.003**	.001**	.027*	.027*	.013*
*ECA*	364 (321–480)	422 (353–459)	318 (257–360)	<.001**	338 (265–419)	360 (315–413)	387 (324–427)	.118	.576	.001**	<.001**	.038*	.033*	.001**
*ICAex*	394 (341–498)	414 (330–495)	291 (190–343)	<.001**	331 (277–424)	367 (311–419)	380 (331–450)	.082	.791	<.001**	<.001**	.004**	.026*	<.001**
*ICAin*	411 (351–501)	453 (315–489)	256 (178–326)	<.001**	347 (288–439)	383 (321–431)	400 (318–433)	.358	.668	<.001**	<.001**	.010*	.102	<.001**
*ACA*	334 (269–370)	317 (230–371)	172(124-229)	<.001**	252 (169–302)	267 (200–305)	287 (239–322)	.084	.444	<.001**	<.001**	<.001**	.020*	<.001**
*MCA*	359 (307–436)	377 (296–465)	215 (149–289)	<.001**	305 (254–372)	340 (270–375)	360 (301–400)	.069	.620	<.001**	<.001**	.003**	.034*	<.001**
*PcomA*	251 (165–324)	248 (220–306)	125 (75–168)	<.001**	154 (96–243)	215 (124–275)	161 (92–216)	.343	.682	<.001**	<.001**	.012*	.054	.009**
*PCA*	292 (255–362)	312 (249–367)	206 (122–245)	<.001**	280 (222–312)	287 (238–337)	295 (248–349)	.212	.953	<.001**	<.001**	.012*	.234	<.001**
*SCA*	188 (157–246)	191 (143–216)	116 (88–173)	<.001**	155 (126–173)	159 (141–230)	160 (133–215)	.171	.397	<.001**	<.001**	<.001**	.538	<.001**
*AICA*	157 (135–194)	166 (135–198)	120 (102–123)	<.001**	139 (115–188)	147 (124–178)	121 (114–143)	.053	.719	<.001**	<.001**	.159	.208	.108
*VA*	363 (291–421)	395 (313–440)	285 (210–311)	<.001**	328 (255–373)	336 (290–393)	338 (282–389)	.716	.491	<.001**	<.001**	.057	.024*	<.001**
*PICA*	199 (144–259)	186 (158–222)	153 (107–173)	<.001**	172 (123–248)	145 (110–202)	182 (129–228)	.261	.430	.001**	.001**	.119	.012*	.061
*AcomA*	245 (197–315)	230 (160–309)	199 (138–223)	.011*	272 (159–294)	223 (154–284)	243 (192–292)	.712	.633	.003**	.028*	.391	.445	.022*
*BA*	358 (316–402)	365 (304–412)	265 (171–336)	.001**	311 (252–367)	341 (262–382)	334 (307–392)	.405	.914	.001**	.001**	.056	.273	.002**

ME data are presented as medians (25th percentile–75th percentile). Overall p-value was calculated by Kruskal-Wallis test. Pairwise comparison was performed by means of Mann-Whitney U-test with interim analyses by Bonferroni-Holm adjustment. *A p-value<0.05 was considered statistically significant. ** p<0.01.

**Table 5:** Calculated SNR values in the groups receiving contrast medium tailored to patient body weight.

					*Paired comparison (p-value)*					
*Vascular territory*	*Group 1 (1.5 ml/kg)*	*Group 2 (1.0 ml/kg)*	*Group 3 (0.5 ml/kg)*	*Multiple comparison (p-value)*	*Group 1/Group 2*	*Group 1/Group 3*	*Group 2/Group 3*	*Group1/Control 1*	*Group 2/Control 2*	*Group 3/Control 3*
*CCA*	65.9 (53.2 – 80.1)	73.5 (58.1 – 83.8)	57.7 (41.4 – 70.8)	.003**	.240	.020*	.001**	.019*	.150	.124
*ECA*	66.5 (51.4 – 80.2)	72.5 (59.9 – 81.7)	55.3 (36.6 – 68.7)	<.001**	.267	.005**	<.001**	.006**	.176	.067
*ICAex*	70.9 (53.8 – 83.0)	74.1 (56.3 – 85.3)	52.5 (26.9 – 61.4)	<.001**	.633	<.001**	<.001**	<.001**	.349	.003**
*ICAin*	71.0 (58.5 – 84.5)	75.6 (52.1 – 85.1)	47.2 (27.5 – 58.4)	<.001**	.791	<.001**	<.001**	.002**	.342	<.001**
*ACA*	54.6 (41.9 – 68.0)	55.5 (39.6 – 69.1)	30.3 (16.9 – 36.1)	<.001**	.590	<.001**	<.001**	<.001**	.356	<.001**
*MCA*	61.3 (51.7 – 73.9)	64.4 (51.3 – 81.8)	40.1 (19.5 – 45.1)	<.001**	.573	<.001**	<.001**	<.001**	.232	<.001**
*PcomA*	43.1 (28.4 – 55.4)	46.0 (31.6 – 52.1)	20.9 (12.9 – 27.3)	<.001**	.922	<.001**	<.001**	.020*	.098	.001**
*PCA*	52.9 (42.6 – 63.4)	54.7 (40.8 – 66.9)	37.8 (18.9 – 42.9)	<.001**	.999	<.001**	<.001**	<.001**	.380	<.001**
*SCA*	32.3 (27.5 – 40.5)	30.3 (25.9 – 38.9)	23.8 (13.9 – 26.0)	<.001**	.361	<.001**	<.001**	<.001**	.211	<.001**
*AICA*	24.2 (18.8 – 29.2)	25.9 (19.6 – 37.3)	22.4 (16.4 – 29.1)	.007*	.351	.043*	.004**	.028*	.055	.123
*VA*	64.0 (48.2 – 73.6)	69.0 (54.5 – 78.5)	51.5 (31.4 – 59.9)	<.001**	.386	<.001**	<.001**	.002**	.050	.041*
*PICA*	29.0 (25.0 – 39.7)	32.7 (26.8 – 37.0)	25.4 (19.4 – 34.7)	.022*	.547	.028*	.010*	.234	.218	.843
*AcomA*	40.8 (35.2 – 55.4)	44.4 (30.8 – 55.3)	32.3 (22.4 – 36.6)	.006**	.747	.002**	.012*	.124	.761	.128
*BA*	62.3 (52.2 – 76.6)	65.3 (49.3 – 76.5)	48.1 (23.2 – 57.7)	.001**	.914	.002**	.001**	.011*	.518	.062

SNR data are presented as medians (25th percentile–75th percentile). Overall p-value was calculated by Kruskal-Wallis test. Pairwise comparison was performed by means of Mann-Whitney U-test with interim analyses by Bonferroni-Holm adjustment. *A p-value<0.05 was considered statistically significant. ** p<0.01.

**Table 6:** Calculated CNR values in the groups receiving contrast medium tailored to patient body weight.

					*Paired comparison (p-value)*					
*Vascular territory*	*Group 1 (1.5 ml/kg)*	*Group 2 (1.0 ml/kg)*	*Group 3 (0.5 ml/kg)*	*Multiple comparison (p-value)*	*Group 1/Group 2*	*Group 1/Group 3*	*Group 2/Group 3*	*Group1/Control 1*	*Group 2/Control 2*	*Group 3/Control 3*
*CCA*	59.0 (46.0 – 73.8)	65.9 (52.9 – 75.6)	50.7 (36.4 – 63.6)	.003**	.269	.018*	.001**	.019*	.173	.098
*ECA*	59.7 (45.6 – 73.8)	65.2 (53.9 – 73.9)	48.4 (31.2 – 60.3)	<.001**	.320	.004**	<.001**	.006**	.254	.051
*ICAex*	65.0 (48.1 – 75.9)	66.3 (50.3 – 77.3)	47.0 (22.5 – 52. 6)	<.001**	.675	<.001**	<.001**	<.001**	.367	.001**
*ICAin*	64.4 (50.9 – 77.7)	68.5 (46.1 – 78.0)	39.3 (21.7 – 51.3)	<.001**	.787	<.001**	<.001**	.002**	.401	<.001**
*ACA*	47.3 (35.8 – 60.5)	48.0 (32.9 – 61.2)	24.5 (11.2 – 32.6)	<.001**	.560	<.001**	<.001**	<.001**	.333	<.001**
*MCA*	55.4 (46.2 – 67.1)	58.0 (45.9 – 74.6)	33.1 (13.7 – 39.6)	<.001**	.586	<.001**	<.001**	<.001**	.274	<.001**
*PcomA*	35.7 (22.8 – 49.2)	38.9 (26.0 – 44.6)	14.6 (6.7 – 20.1)	<.001**	.922	<.001**	<.001**	.018*	.067	.001**
*PCA*	46.3 (36.3 – 57.7)	47.8 (34.7 – 59.2)	31.2 (13.3 – 35.7)	<.001**	.961	<.001**	<.001**	<.001**	.383	<.001**
*SCA*	26.3 (20.5 – 34.8)	22.7 (19.5 – 31.7)	15.6 (9.3 – 19.4)	<.001**	.341	<.001**	<.001**	<.001**	.202	<.001**
*AICA*	18.2 (12.6 – 23.0)	19.4 (13.3 – 29.8)	12.1 (8.2 – 19.0)	.014*	.390	.037*	.004**	.039*	.059	.076
*VA*	58.3 (41.2 – 66.6)	62.3 (48.8 – 70.7)	45.0 (26.4 – 52.8)	<.001**	.427	<.001**	<.001**	.003**	.061	.033*
*PICA*	22.8 (18.1 – 33.4)	25.6 (20.1 – 29.5)	18.9 (12.6 – 27.2)	.012*	.665	.018*	.005**	.331	.309	.735
*AcomA*	34.9 (29.5 – 48.0)	36.5 (24.5 – 47.6)	25.2 (16.5 – 30.0)	.006**	.715	.002**	.016*	.162	.867	.123
*BA*	54.8 (45.5 – 70.3)	58.0 (43.1 – 69.0)	42.2 (17.5 – 48.9)	.001**	.978	.001**	.001**	.012*	.593	.050

CNR data are presented as medians (25th percentile–75th percentile). Overall p-value was calculated by Kruskal-Wallis test. Pairwise comparison was performed by means of Mann-Whitney U-test with interim analyses by Bonferroni-Holm adjustment. *A p-value<0.05 was considered statistically significant. ** p<0.01.

### Paired Comparison of Body Weight-Tailored Groups

Comparison of ME, SNR, and CNR between group 1 (1.5 ml/kg) and group 2 (1.0 ml/kg) revealed no significant differences in any vascular territories (Tables 4, 5 and 6). Comparison of group 1 (1.5 ml/kg) and group 3 (0.5 ml/kg) showed statistically higher ME, SNR, and CNR in all vascular territories for group 1 (1.5 ml/kg). The paired comparison of group 2 (1.0 ml/kg) and group 3 (0.5 ml/kg) yielded similar results with significantly higher ME, SNR, and CNR in group 2 (1.0 ml/kg).

### Paired Comparison of Body Weight-Tailored Groups versus Standard Protocol

Paired comparison of the weight-tailored groups versus the standard protocol regarding ME, SNR and CNR yielded heterogeneous results. Group 1 (1.5 ml/kg) had higher values compared to the standard protocol for the vascular territories of the anterior circulation (CCA, ECA, ICAex, ICAin, ACA, MCA, not AcomA) and the upper vessels of the posterior circulation (PCA, PcomA, SCA) regarding ME, SNR, and CNR (Tables 4, 5 and 6). For the remaining vessels of the posterior circulation, there was no statistically significant difference in ME, but SNR and CNR for AICA, VA and BA were also higher for group 1 (1.5 ml/kg) (Tables 5 and 6). Group 2 (1.0 ml/kg) had higher ME in the CCA, ECA, ICAex, ACA, MCA, VA, and PICA (Table 4), but SNR and CNR were not higher (Tables 5 and 6). Regarding group 3 (0.5 ml/kg), the standard protocol yielded better results for ME in all vascular territories except AICA and PICA (Table 4), whereas SNR and CNR failed to reach significance for CCA, ECA, AICA, PICA, AcomA, and BA (Tables 5 and 6).

### Relationship between Patient Body Weight/BMI and ME in the MCA

**Figure 1:**
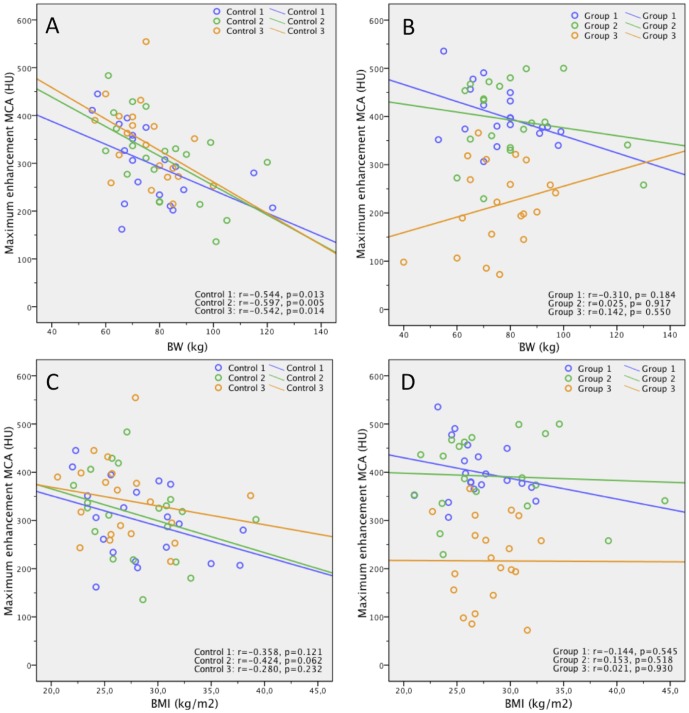
Correlation of body weight/BMI and ME in the MCA. Scatterplots show relationship between patient body weight (BW) (kg) as well as BMI (kg/m^2^) and ME of MCA (HU) for the three controls (A, C) and for the three groups (B, D). Lines are regression lines. Spearman's rank correlation coefficients (r) and p-values are provided in the lower right corner.

Figure 1 presents scatterplots of the relationship between patient body weight as well as BMI and ME in the MCA for the controls and the body weight-tailored groups. For the control groups, Spearman's rank correlation coefficient (r) between body weight and ME of the MCA was r = −0.544 (p = 0.013) for control 1, r = −0.597 (p = 0.005) for control 2, and r = −0.542 (p = 0.014) for control 3, indicating moderate statistically significant inverse correlation in all three controls. Values for the correlation of BMI and ME in the control groups were comparable with r = −0.358 (p = 0.121) for control 1, r = −0.424 (p = 0.062) for control 2, and r = −0.280 (p = 0.232) for control 3. For the body weight-tailored groups, Spearman's rank correlation coefficient (r) between body weight and ME of the MCA was r = −0.310 (p = 0.184) for group 1 (1.5 ml/kg), r = 0.025 (p = 0.917) for group 2 (1.0 ml/kg), and r = 0.142 (p = 0.550) for group 3 (0.5 ml/kg), indicating a weak inverse correlation for group 1 (1.5 ml/kg) and a very weak direct correlation for group 2 (1.0 ml/kg) and group 3 (0.5 ml/kg) with no statistical significance for any of the three groups. Similar values were found for the correlation of BMI and ME in the body weight-tailored groups with r = −0.144 (p = 0.545) for group 1 (1.5 ml/kg), r = 0.153 (p = 0.518) for group 2 (1.0 ml/kg) and r = 0.021 (p = 0.930) for group 3 (0.5 ml/kg).

### BMI≤25 and BMI>25 Subgroup Analysis for ME of the MCA

**Figure 2:**
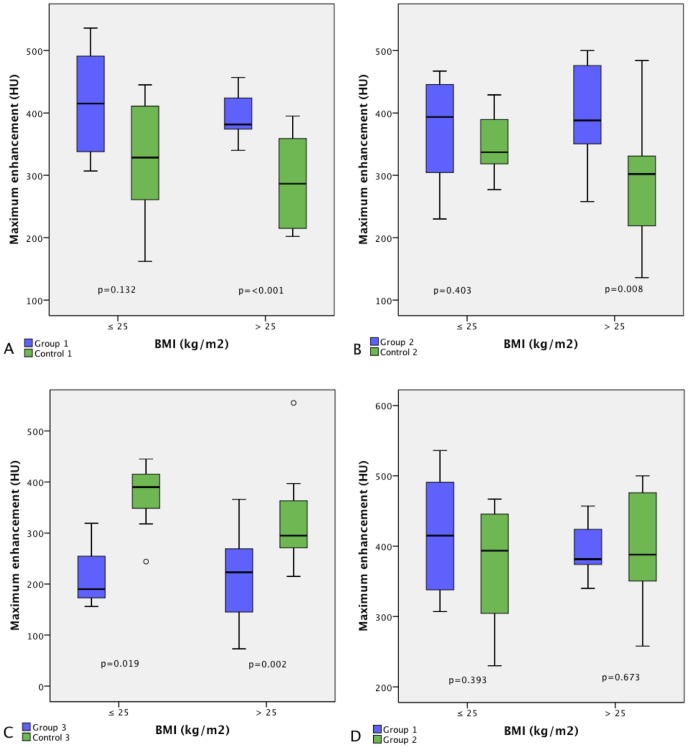
Comparison of ME in the MCA between BMI≤25 and BMI>25 subgroups. Boxplots compare ME in the MCA in groups and controls (A–C) and between group 1 (1.5 ml/kg) and group 2 (1.0 ml/kg) (D) in normal weight patients (BMI≤25) and overweight patients (BMI>25). In boxes, middle horizontal line and upper and lower margins represent median with 25th and 75th percentiles. Upper and lower ends of vertical lines represent upper extremes (75th percentile +1.5× [interquartile range]) and lower extremes (25th percentile−1.5× [interquartile range]), respectively.

Figure 2 illustrates ME in the MCA for groups and controls subdivided by BMI into a normal weight group (BMI≤25) and an overweight group (BMI>25). The higher ME in the MCA for group 1 (1.5 ml/kg) and group 2 (1.0 ml/kg) compared with the standard protocol was not statistically significant for patients with normal body weight (Fig. 2 A, B; p = 0.132/p = 0.403). In contrast, overweight patients in both groups had statistically significant higher ME compared to the standard protocol (Fig. 2 A, B; p = <0.001/p = 0.008). Comparison of the ME in the overweight subgroups of group 1 (1.5 ml/kg) and group 2 (1.0 ml/kg) yielded no statistically significant difference (Fig. 2 D; p = 0.673). The lower ME in the MCA in group 3 (0.5 ml/kg) compared with control 3 was statistically significant for both subgroups (Fig. 2 C; p = 0.019/p = 0.002).

### Qualitative Visual Assessment

**Figure 3:**
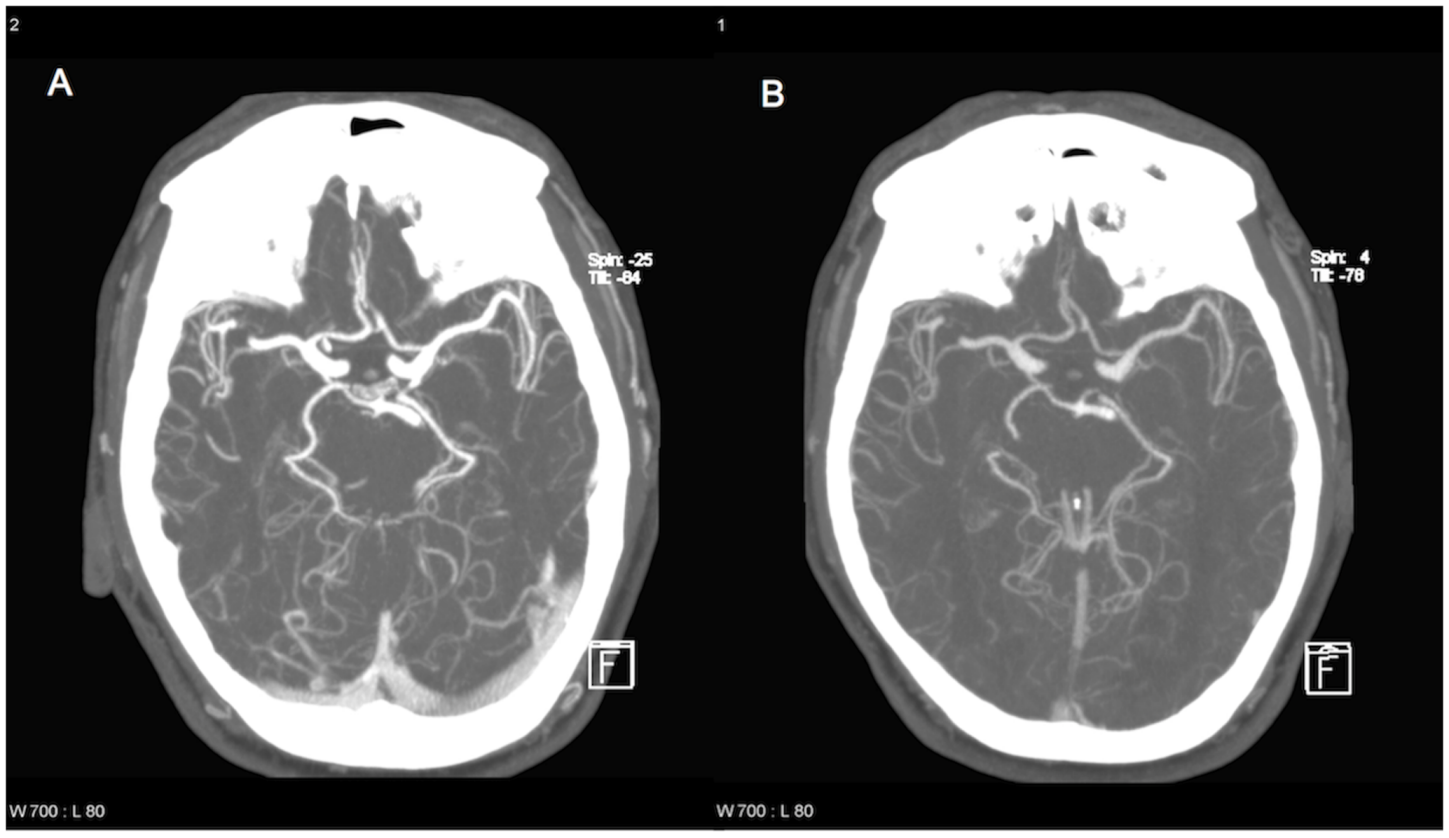
Sample images for comparison of image quality between group 2 and group 3. By chance, a 56-year-old male patient (86 kg) was examined twice during the study period. Both images show thick slab maximum intensity projections (MIP) of corresponding slices. He was examined with 1.0 ml/kg (A, 86 ml CM) and 0.5 ml/kg (B, 43 ml CM). Image A was graded optimal, while image B was graded good by both raters. Arterial enhancement was 318 HU/203 HU for the MCA on the left with 1.0/0.5 ml/kg.

Figure 3 illustrates the difference in image quality between group 2 (1.0 ml/kg) and group 3 (0.5 ml/kg), presenting an example of the reconstructions used for visual assessment. Table 7 presents the results of visual assessment of overall image quality for each of the two readers. Interrater reliability was substantial with κ = 0.68, κ = 0.66, and κ = 0.71 in groups 1 (1.5 ml/kg), 2 (1.0 ml/kg), and 3 (0.5 ml/kg), respectively, and moderate to substantial with κ = 0.55, κ = 0.58, and κ = 0.75 in controls 1, 2, and 3, respectively. For the two raters, overall differences between the three groups were statistically significant with p = 0.002 and p = 0.005, respectively. Paired comparison of image quality in the three groups revealed no statistically significant differences between group 1 (1.5 ml/kg) and group 2 (1.0 ml/kg) for either rater (p = 0.513/p = 0.518) but considerable differences between group 2 (1.0 ml/kg) and group 3 (0.5 ml/kg) (p = 0.001/p = 0.003) and group 1 (1.5 ml/kg) and group 3 (0.5 ml/kg) (p = 0.006/p = 0.01) for both raters. Paired comparisons of image quality in groups and controls showed no statistically significant differences between group 2 (1.0 ml/kg) and control 2 for either rater (p = 0.221/p = 0.383). For group 1 (1.5 ml/kg) versus control 1, rater 1 found significant differences in image quality (p = 0.01) but rater 2 did not (p = 0.512). The situation was reversed for group 3 (0.5 ml/kg) versus control 3 with significant differences in image quality for rater 2 (p = 0.038) but not for rater 1 (p = 0.121). However, overall differences between the three controls were not statistically significant with p = 0.142 for rater 1 and p = 0.831 for rater 2.

**Table 7:** Results of visual assessment of overall image quality.

*Rater*	*1*					*2*				
	*Unacceptable*	*Poor*	*Fair*	*Good*	*Optimal*	*Unacceptable*	*Poor*	*Fair*	*Good*	*Optimal*
*Group 1*	0	0	0	8 (40)	12 (60)	0	0	0	7 (35)	13 (65)
*Group 2*	0	0	0	6 (30)	14 (70)	0	0	1 (5)	7 (35)	12 (60)
*Group 3*	0	2 (10)	3 (15)	7 (35)	8 (40)	0	2 (10)	3 (15)	9 (45)	6 (30)
*Control 1*	0	0	6 (30)	9 (45)	5 (25)	0	0	4 (20)	7 (35)	9 (45)
*Control 2*	0	1 (5)	1 (5)	8 (40)	10 (50)	0	1 (5)	1 (5)	9 (45)	9 (45)
*Control 3*	0	1 (5)	2 (10)	7 (35)	10 (50)	0	1 (5)	2 (10)	6 (30)	11 (55)

Results are given as total numbers of patients assigned to each quality category. Numbers in brackets are percentages.

Data are given by rater and protocol group.

## Discussion

Generally, the degree of arterial enhancement in CTA is determined by injection-related factors such as CM volume, CM concentration, and injection rate and by patient-related factors such as body weight, cardiac output, and fluid balance [19].

In our study, different CM volumes were tailored to the patients' body weight, whereas CM concentration and injection rate were constant. We used three dose groups of 1.5 ml/kg, 1.0 ml/kg, and 0.5 ml/kg with a CM concentration of 270 mg iodine/ml and a fixed injection rate of 4 ml/s.

The variability in arterial enhancement resulting from differences in cardiac output can be reduced by administering a test bolus or using a bolus-tracking system [20]–[22]. To minimize the effects of cardiac output in the present study, we used a semiautomatic bolus-tracking system and excluded patients assumed to have compromised cardiac output.

Furthermore, vessel stenoses and occlusions can influence the degree of contrast enhancement in downstream vessel territories. Patients undergoing craniocervical CTA often have vessel stenoses or occlusions in the CCA, ICA, MCA, VA, or BA. In our study population, several patients presented with occlusion or stenosis of the carotid artery, vertebral artery, and basilar artery. While these lesions were excluded from evaluation, we nevertheless took measurements in the vessel territories distal to the lesions. The measured values were compared with the ME for the corresponding contralateral measurement site. The comparison revealed no statistically significant differences in ME, which is most probably due to good collateralization in chronic stenosis or occlusion [23], [24].

An inverse correlation is known to exist between patient body weight and arterial or parenchymal enhancement in thoracic or abdominal CT when a constant dose of CM is used [3], [6], [11], [16], [25], [26]. Our study illustrates that a moderate inverse correlation between body weight and BMI and maximum arterial enhancement is also found in craniocervical CTA performed with a standard dose of 80 ml of Visipaque 270® (Fig. 1 A, C).

Compared to the standard protocol with use of 80 ml of Visipaque 270®, two of the three tailored protocols −1.0 ml/kg and 1.5 ml/kg - yielded similar or better results with regard to ME, along with excellent or good image quality. Compared with each other, the two higher doses were comparable in terms of ME, SNR, and CNR, whereas both provided significantly better ME, SNR, and CNR as well as image quality than 0.5 ml/kg. However, administration of 1.5 ml/kg mainly improved arterial enhancement of the cervical vessels and circle of Willis, whereas significant effects on the posterior circulation were only detectable for SNR and CNR in the VA and BA. The dose of 1.0 ml/kg produced significantly higher ME in the cervical vessels, the ACA, MCA, VA, and PICA, whereas differences to the standard protocol were not significant for SNR and CNR. For the dose of 0.5 ml/kg, ME, SNR, and CNR were mainly inferior to the standard protocol and especially to the body weight-adjusted doses of 1.0 ml/kg and 1.5 ml/kg. This effect might be due to substantial dilution of the small CM bolus in the large and medium-sized cerebral arteries compared to the standard dose and higher weight-adjusted doses. In the small cerebral arteries (AcomA, PICA, and AICA), the difference in ME, SNR and CNR between 0.5 ml/kg and the standard dose is no longer significant. Presumably, the effect of a larger contrast medium bolus is particularly evident in the large cervical and intracranial arteries whereas the bolus dilution in the smaller cerebral arteries is similar in all groups independent of the initial bolus size.

In overweight patients (BMI>25), the body weight-tailored doses of 1.0 ml/kg and 1.5 ml/kg produce significantly higher ME compared to the standard dose, but compared to each other, the highest dose of 1.5 ml/kg does not result in higher ME than the dose of 1.0 ml/kg. In normal weight patients (BMI≤25), the tailored doses of 1.0 ml/kg and 1.5 ml/kg yield similar enhancement as the standard dose.

## Conclusion

Administration of 1.0 ml/kg of CM, corresponding to 270 mg iodine/kg, can be recommended for craniocervical CTA.

In patients weighing less than 80 kg, the dose of 1.0 ml/kg reduces the iodine dose compared to a standard dose of 80 ml, while yielding similar arterial enhancement. A reduced dose of CM has potential benefits: first, the likelihood of CIN decreases, particularly in patients with renal insufficiency [27]. Second, if digital subtraction angiography becomes necessary, e.g., for a neurovascular intervention, immediately after craniocervical CTA, a restriction of the CM dose is favorable for preventing high loads of CM. Third, a reduced CM volume results in cost savings.

For overweight patients (BMI>25) the body weight-adjusted dose of 1.0 ml/kg improves arterial contrast. A threshold of 150 kg should be considered for tailoring the contrast medium dose to body weight to adhere to the FDA label for Visipaque 270® (maximum total volume of 150 ml for contrast-enhanced CT of head or body)[28].

### Study Limitations

Our study has some limitations. First, Kondo et al. and Ho et al. show, for abdominal CT, that if lean body weight or body fat percentage rather than body weight are used to determine the appropriate CM dose, the iodine dose required to achieve sufficient enhancement may be estimated more precisely [29]–[31]. Hence, further studies will be necessary to determine the optimal parameter for individually tailored CM administration for craniocervical CTA. Second, patients were consecutively assigned to the protocol groups and not randomized because of ethical concerns regarding the possible nondiagnostic image quality related to a reduced dose. The study was started with the highest dose, which was consecutively reduced but only after verification of image quality before each reduction. Third, contrast enhancement was measured not only in cervical and large cerebral arteries but also in the smaller and more variable cerebral arteries, e.g., AcomA, PcomA, PICA, and AICA. On the one hand, measurement is technically more demanding in these vascular territories because partial volume effects reduce the reliability of data, and on the other hand, the number of measured values is reduced due to anatomical variations.
